# Supplementary figures and images for: The planarian dorsal–ventral boundary regulates anterior–posterior axis growth and patterning
Source: PLoS Biol. 2025 Nov 11;23(11):e3003482. doi: 10.1371/journal.pbio.3003482 (PMC12629446; doi:10.1371/journal.pbio.3003482)

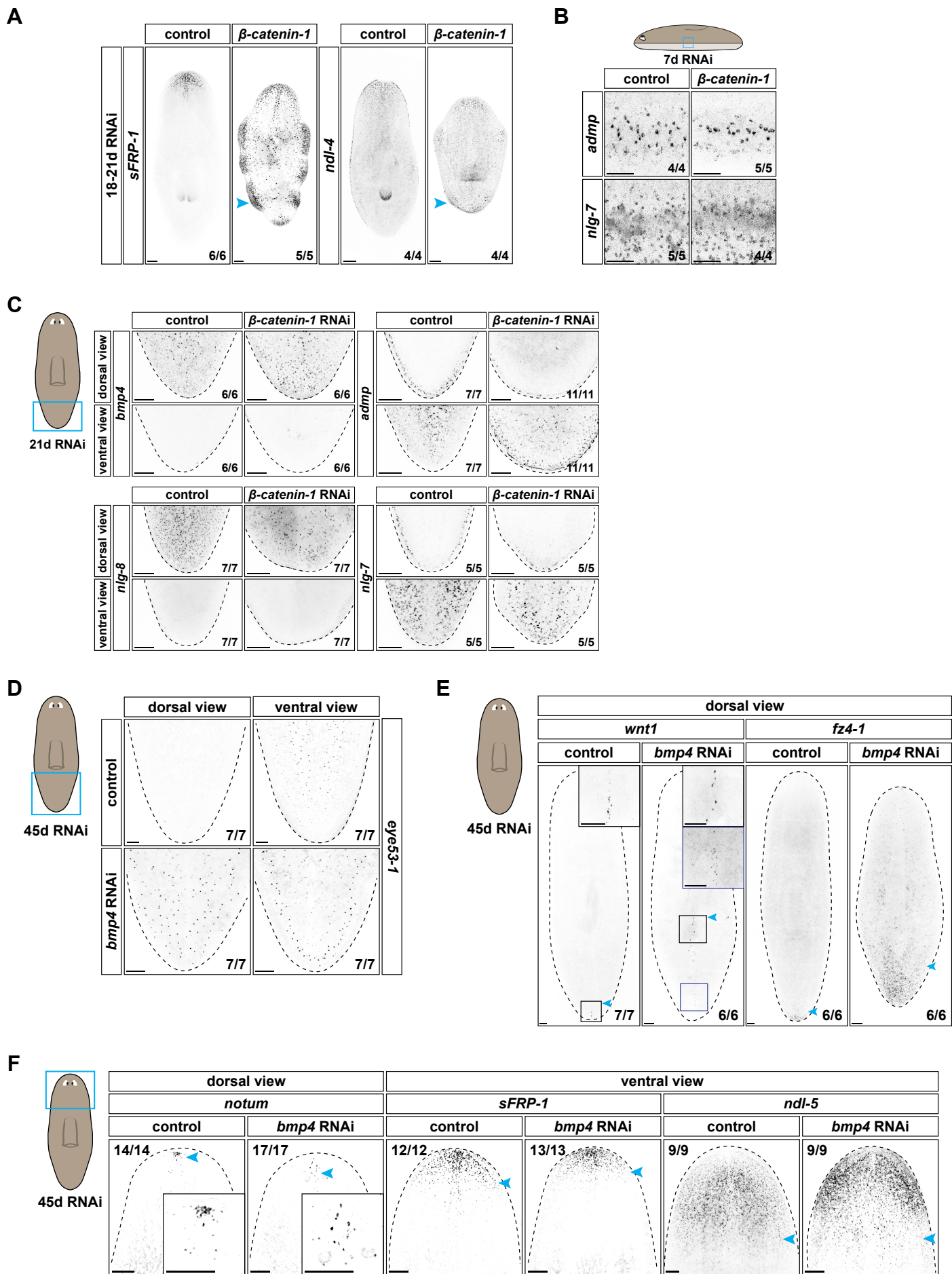

S1 Fig

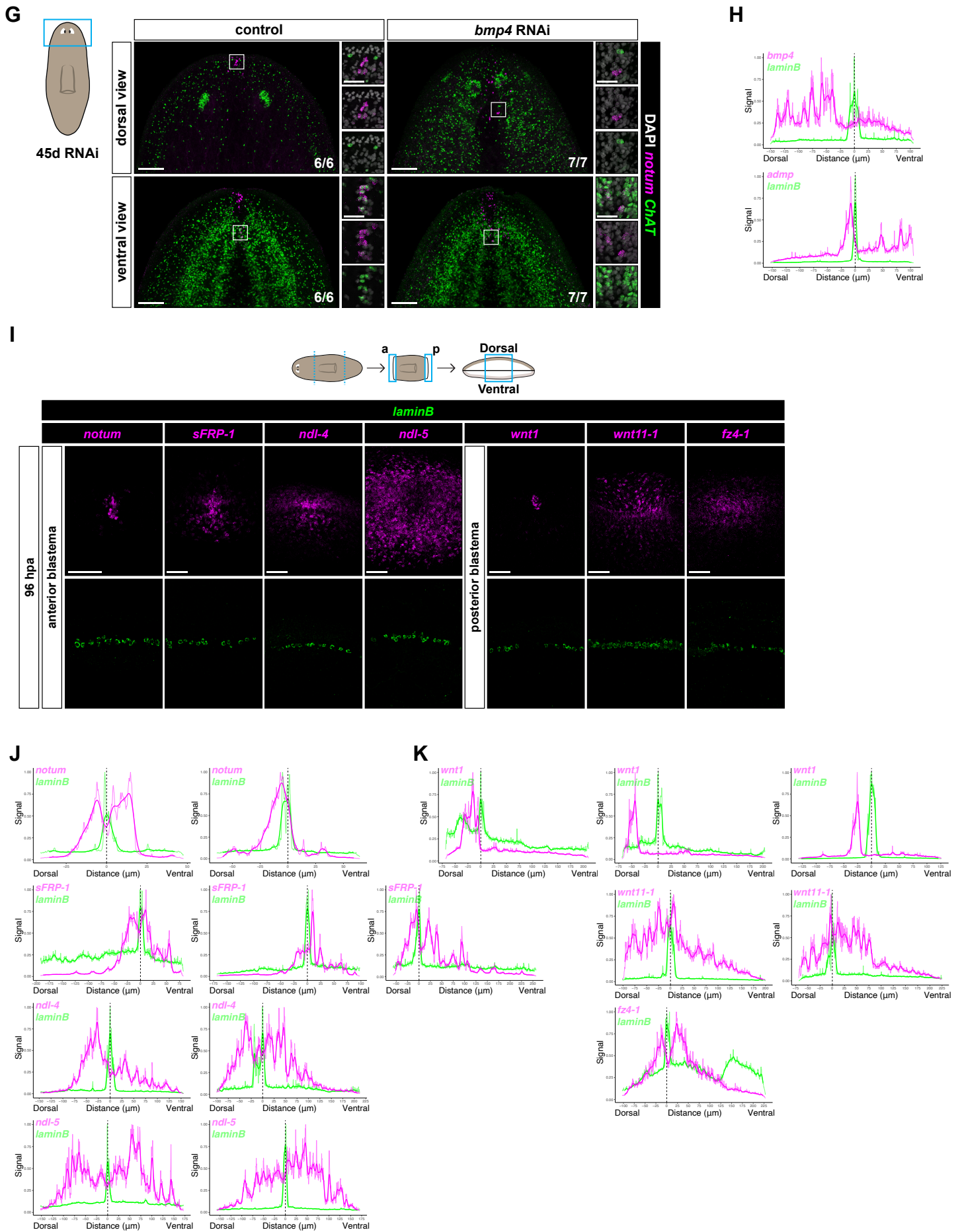

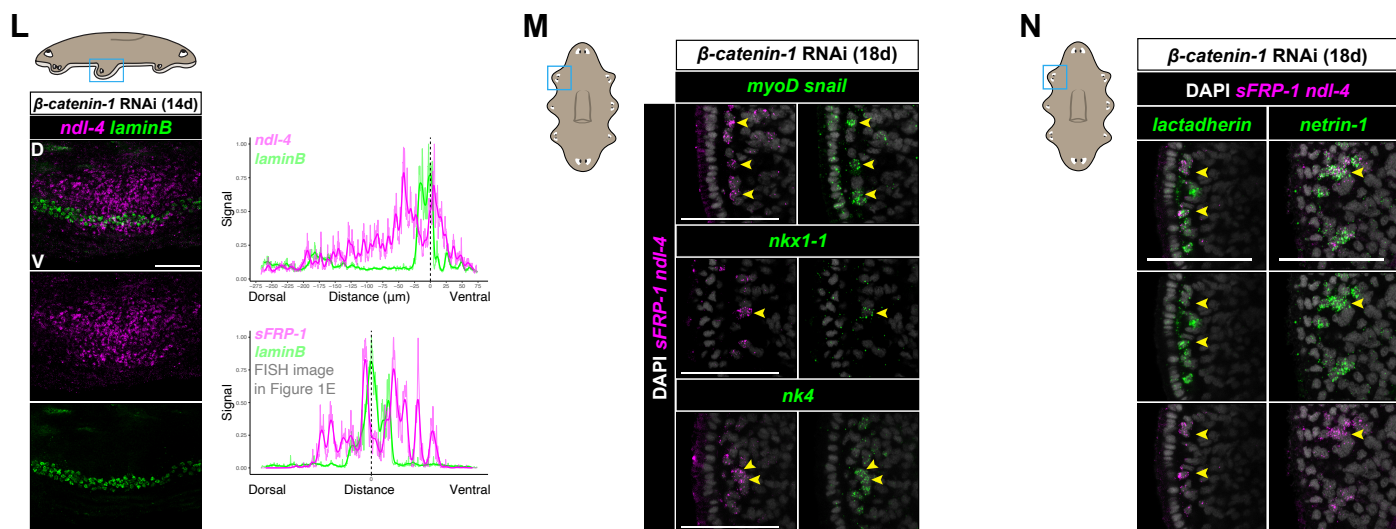

Supplement: S1 Fig — A) Posterior-lateral expression of anterior PCGs (sFRP-1, ndl-4) following β-catenin-1 RNAi (18–21d). Foci containing multiple anterior PCG+ cells were apparent at the DVB (foci typically had >10 cells). Scattered anterior PCG+ cells were also present in the animal. B) Ventrally biased (admp, nlg-7) PCG expression at the DVB after β-catenin-1 RNAi (7d). C) Dorsally biased (bmp4, nlg-8) and ventrally biased (admp, nlg-7) PCG expression in tails after β-catenin-1 RNAi (21d). D) Dorsal expression of the ventral neural marker eye53-1 following bmp4 RNAi (45d). E, F) PCG expression domains in bmp4 RNAi animals (45d). Blue arrow denotes anterior (posterior PCG) or posterior (anterior PCG) boundary of PCG expression domain. G) The posterior expansion of notum signal on the dorsal surface after bmp4 RNAi is because of expansion of the anterior pole (notum+ChAT−). H) Fluorescence intensity of the PCGs bmp4 and admp and the epidermal DVB marker laminB along a line is plotted. Smoothed data is represented with a thick stroke. Individual data points are listed in S1 Data. I) Anterior and posterior PCG expression is concentrated near the DVB during regeneration (96 hpa). Single-channel images of the merged images in Fig 1C are presented. J, K) Fluorescence intensity of anterior and posterior PCGs and the epidermal DVB marker laminB along a line is plotted. Smoothed data is represented with a thick stroke. Individual data points are listed in S1 Data. L) Ectopic anterior PCG (ndl-4) expression near the DVB after β-catenin-1 RNAi (14d). D, dorsal; V, ventral. Fluorescence intensity along a line is plotted and individual data points are listed in S1 Data. M) Ectopic anterior PCG (sFRP-1, ndl-4) expression after β-catenin-1 RNAi (18d) is detected in longitudinal (myoD+), circular (nkx1-1+), and lateral DV (nk4+) muscle at the lateral animal edge. Single-channel images of the merged images in Fig 1F are presented. N) Co-expression of anterior PCGs (sFRP-1, ndl-4) and the secreted mol [file pbio.3003482.s001.pdf]

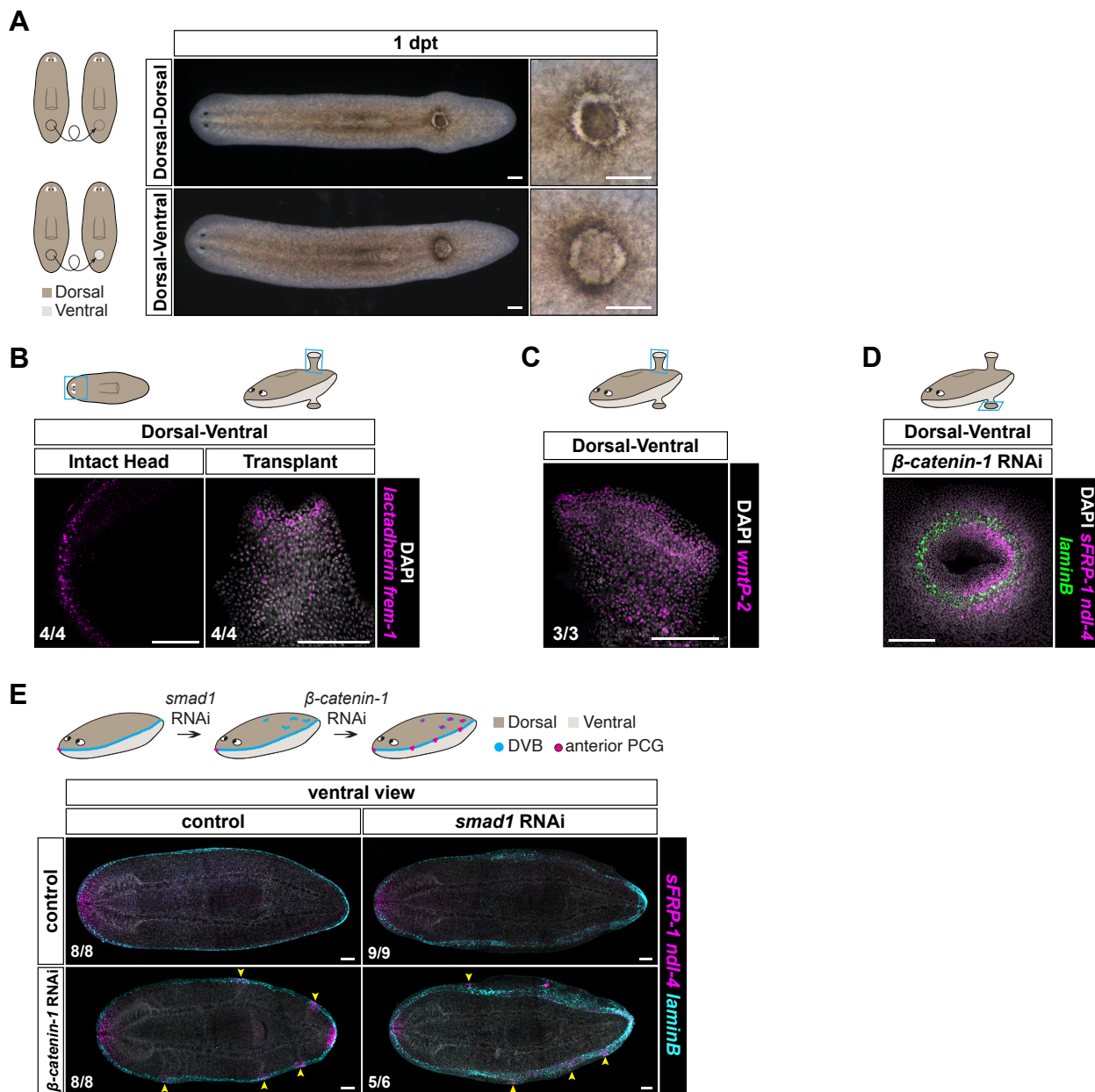

Supplement: S3 Fig — A) D-V transplant animals one day post-transplant (dpt). B) D-V transplant outgrowths show muscle DVB gene expression (lactadherin, frem-1). C) Post-pharyngeal D-V transplant outgrowths express wntP-2, indicative of posterior identity. D) Ventral outgrowths that result from D-V transplant are anteriorized (sFRP-1 ndl-4+) after β-catenin-1 RNAi (10–14d). E) smad1 RNAi animals retain their original DVB (laminB), which is anteriorized (sFRP-1 ndl-4+) after β-catenin-1 RNAi (10d). Yellow arrowhead, anterior PCG focus. Scale bars, 250 μm (A), 100 μm (B–E). (PDF) [file pbio.3003482.s003.pdf]

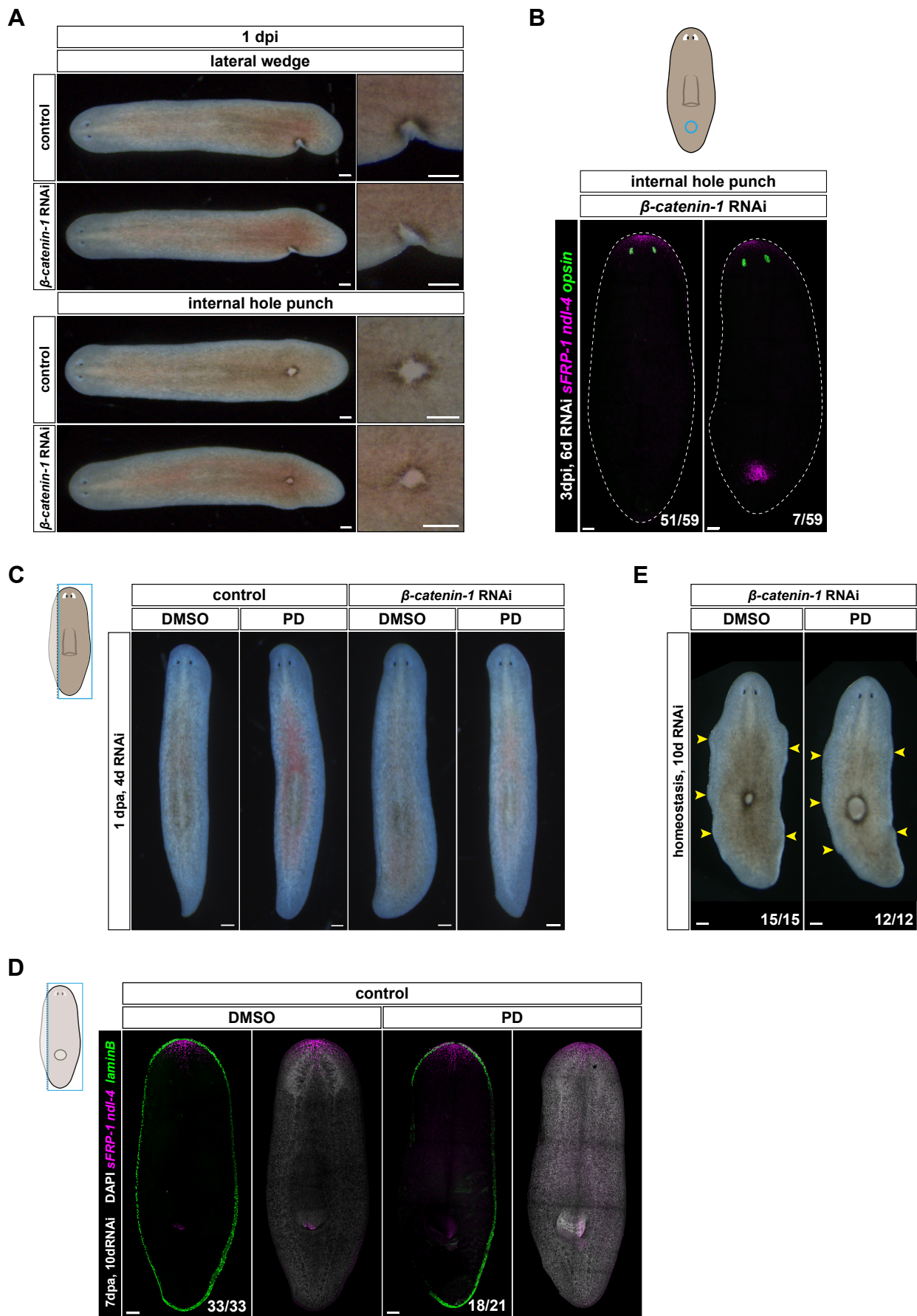

F

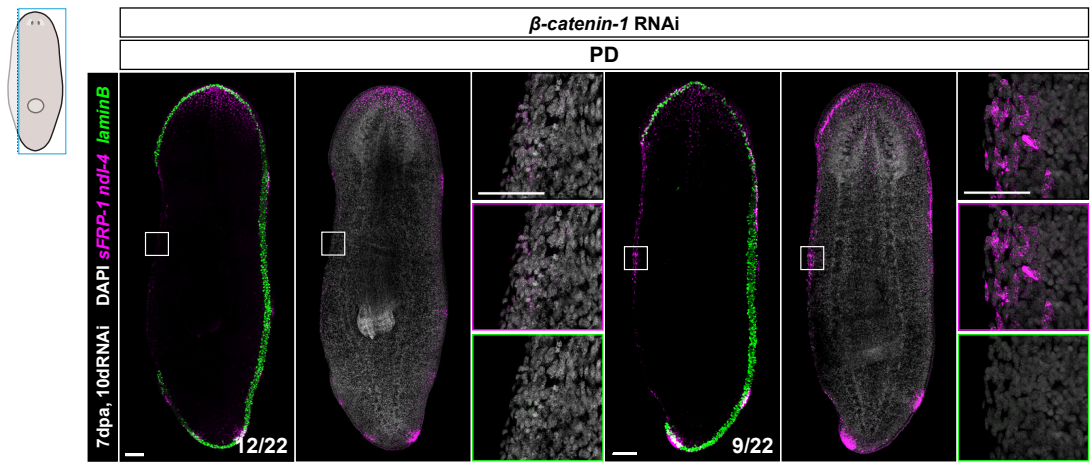

G

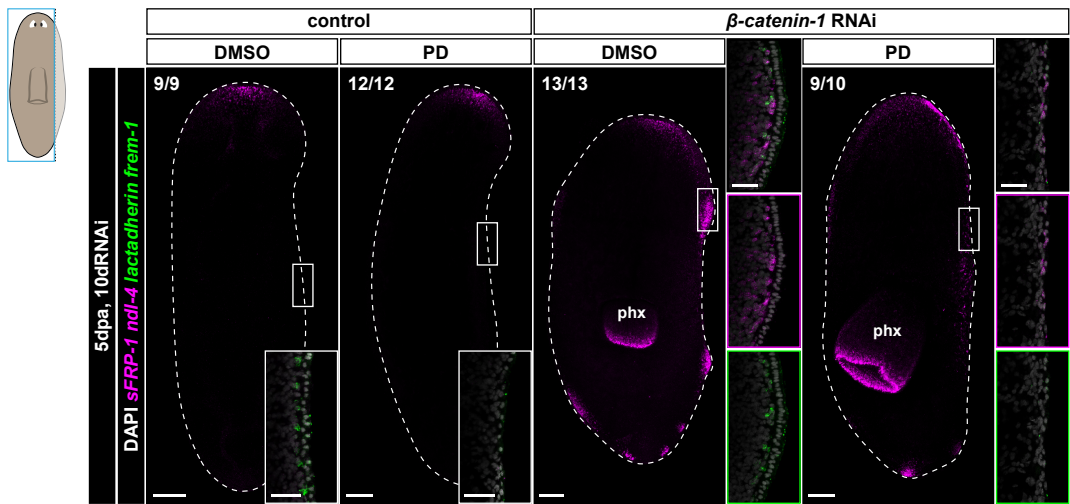

Supplement: S4 Fig — A) Lateral wedge and internal hole punch animals one day post-injury (dpi). B) β-catenin-1 RNAi animals after internal hole punch. In rare cases (n = 7/59), a focus of anterior PCG (sFRP-1, ndl-4) expression develops at the wound. β-catenin-1 RNAi, hole punch animals that do not ectopically express anterior PCGs at the wound (n = 51/59) are shown for comparison and the image from Fig 5A is presented. C) β-catenin-1 RNAi animals incubated in PD one dpa. D) PD treatment blocks regeneration of the DVB (laminB) following parasagittal amputation (n = 18/21). In 3/21 animals, a patch of laminB+ DVB was present after parasagittal amputation and PD treatment, indicating incomplete DVB removal or partial DVB regeneration. E) PD treatment does not prevent homeostatic outgrowth associated with ectopic head formation after β-catenin-1 RNAi (10d). F) β-catenin-1 RNAi animals incubated in PD after parasagittal amputation. In some animals (n = 9/22), anterior PCG (sFRP-1, ndl-4) expression is detected at the wound, though these animals lack PCG foci formation and apparent homeostatic outgrowth. β-catenin-1 RNAi, PD-treated animals that lack anterior PCG expression at the wound (n = 12/22) are shown for comparison and the image from Fig 5B is presented. One animal had a patch of laminB+ DVB present at the wound (n = 1/22), indicating incomplete DVB removal or partial DVB regeneration. G) PD treatment after DVB removal blocks regeneration of muscle DVB (lactadherin, frem-1) cells. Despite no DVB regeneration, low levels of anterior PCG (sFRP-1, ndl-4) expression can be detected at the wound (n = 9/10). Colored boxes, area depicted in photos. Scale bars, 250 μm (A, C, E), 100 μm (B, D, F, G), 50 μm (F, inset), 25 μm (G, inset). (PDF) [file pbio.3003482.s004.pdf]

A

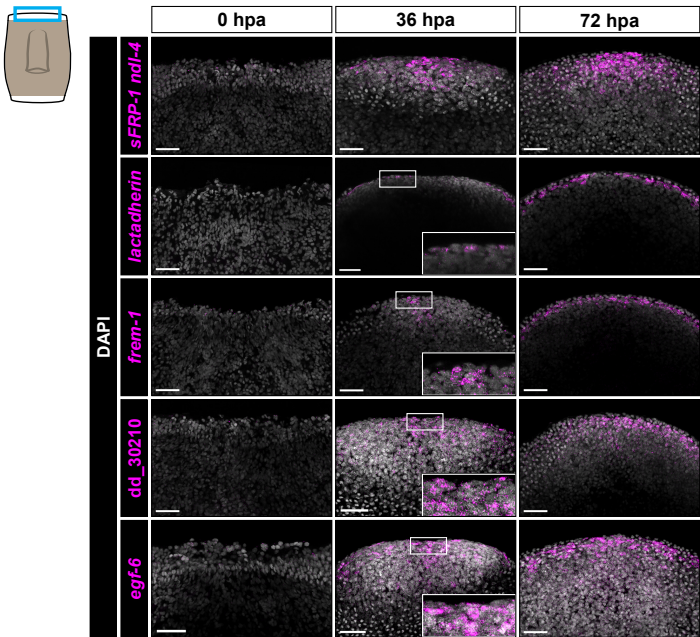

Supplement: S5 Fig — A) Regeneration of anterior PCG (sFRP-1, ndl-4) and muscle DVB (lactadherin, frem-1, dd_30210, egf-6) gene expression domains 0–72 hpa. Patterned muscle DVB gene expression is detected in the blastema by 36 hpa, along with a focus of sFRP-1 ndl-4 expression. Colored box, area depicted in photos. Scale bars, 50 μm (A). (PDF) [file pbio.3003482.s005.pdf]

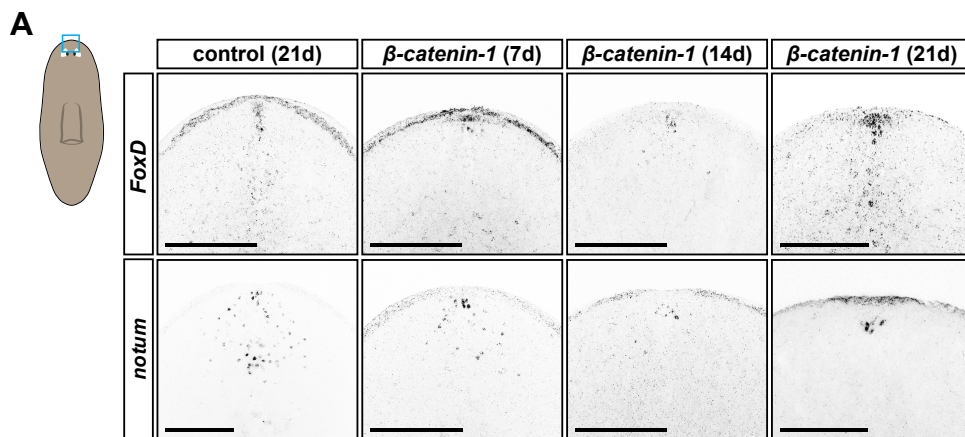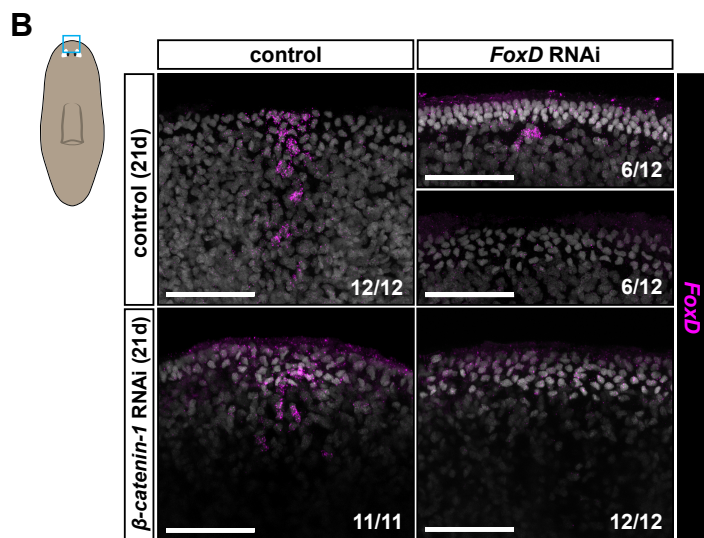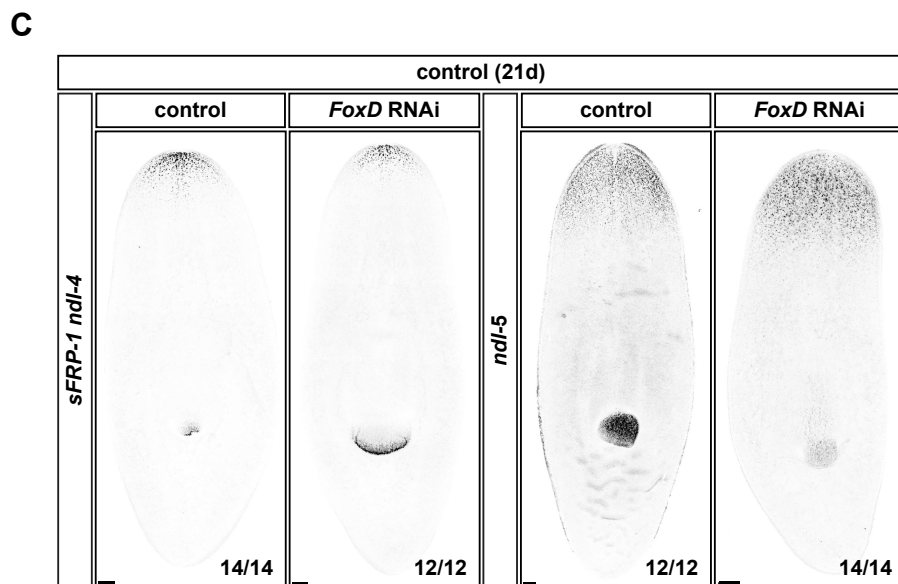

Supplement: S6 Fig — A) Detection of anterior pole transcripts (FoxD, notum) after β-catenin-1 RNAi (7–21d). B) FoxD expression in the anterior pole is significantly reduced (n = 6/12) or absent (n = 6/12) after FoxD; control RNAi and absent (n = 12/12) after FoxD; β-catenin-1 RNAi. C) Anterior PCG (sFRP-1, ndl-4, ndl-5) gradients are not grossly affected by FoxD RNAi. Colored boxes, area depicted in photos. Scale bars, 50 μm (A, B), 100 μm (C). (PDF) [file pbio.3003482.s006.pdf]
