# Supplementary material for: The planarian dorsal–ventral boundary regulates anterior–posterior axis growth and patterning
Source: PLoS Biol. 2025 Nov 11;23(11):e3003482. doi: 10.1371/journal.pbio.3003482 (PMC12629446; doi:10.1371/journal.pbio.3003482)
Supplement: S2 Fig — A) Cartoon shows region of the animal isolated for scRNA-seq. Violin plots show the number of UMIs (left) and genes (center) per cell. Individual data points are listed in S1 Data. Table (right) shows the total number of cells after quality control (QC), mean reads, and mean genes detected per cell. B) Violin plot shows the percentage of reads mapped to mitochondrial genes. Individual data points are listed in S1 Data. C) Subclustering of muscle cells, labeled by colF-2, reveals the muscle subtype composition at the DVB. UMAP plots show the overlapping expression of DVB marker genes in longitudinal muscle cells, labeled by myoD. Novel markers of the DVB are labeled in black. Genes with DVB expression verified by FISH are separated by dotted lines. D) UMAP plots show the expression of DVB marker genes in circular muscle cells. E) Heatmap shows the expression of FISH-screened subcluster 1-enriched genes across myoD+ longitudinal muscle subclusters. F) UMAP plots show DVB-enriched gene expression. G) Table of 32 longitudinal muscle subcluster 1-enriched genes categorized by the type of protein each gene encodes. Genes labeled in bold font have DVB expression patterns verified by FISH. H) Table of 11 unnamed DVB-enriched genes listed by dd_ID. The PFAM domains comprising proteins encoded by these genes are listed. I) Protein domain structure of novel DVB markers. J) Whole-body expression of novel DVB marker genes by FISH. K) Plot shows the Pearson correlation coefficients for pairwise comparisons between DVB-enriched genes in longitudinal muscle subcluster 1. Scale bars, 100 μm (J). (PDF) [file pbio.3003482.s002.pdf]

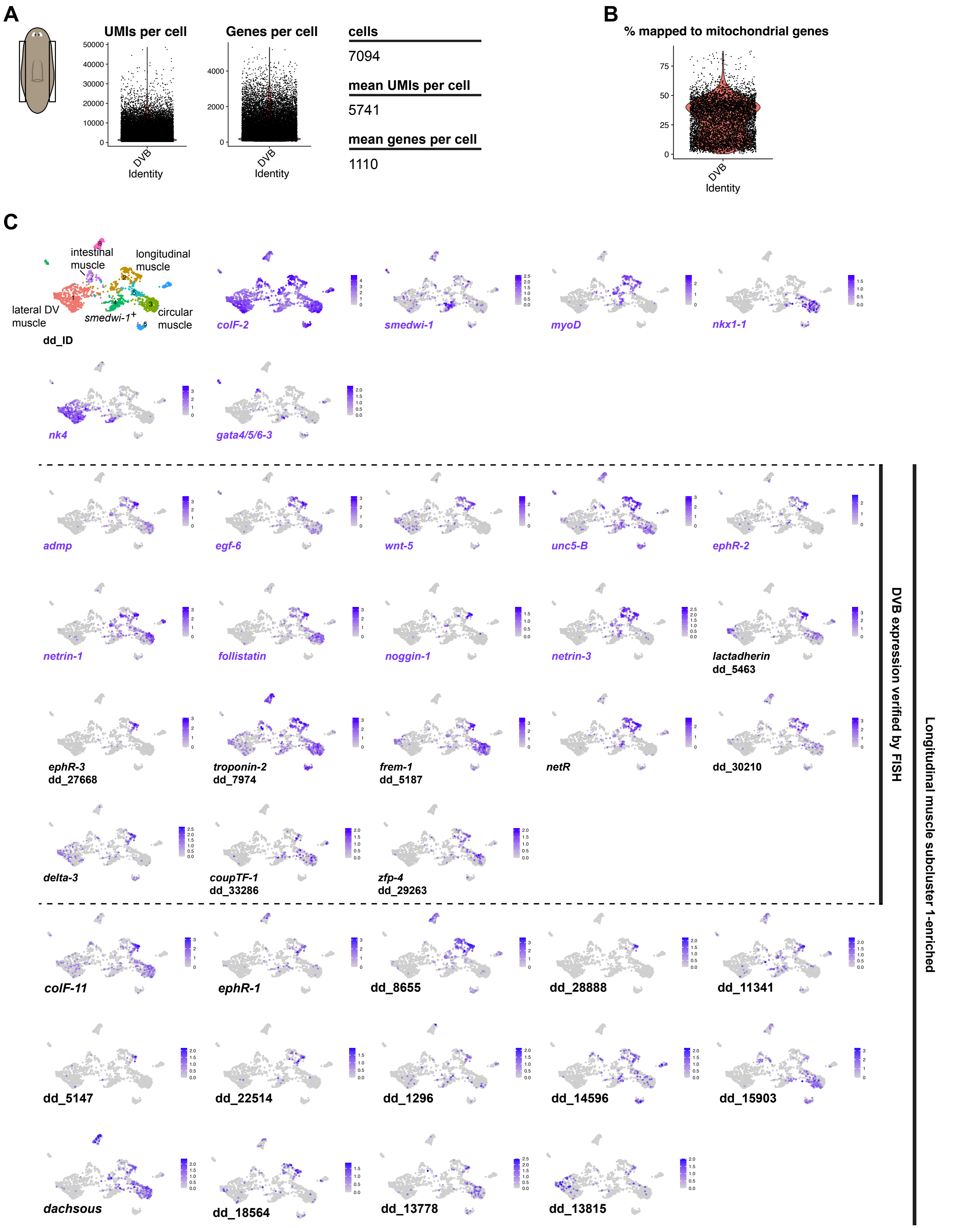
A
UMIs per cell
Genes per cell
DVB
Identity
cells
7094
mean UMIs per cell
5741
mean genes per cell
1110
B
% mapped to mitochondrial genes
C
intestinal muscle
longitudinal muscle
lateral DV muscle
smedwi-1+
circular muscle
dd_ID
colF-2
smedwi-1
myoD
nkx1-1
nk4
gata4/5/6-3
admp
egf-6
wnt-5
unc5-B
ephR-2
netrin-1
follistatin
noggin-1
netrin-3
lactadherin dd_5463
ephR-3 dd_27668
troponin-2 dd_7974
frem-1 dd_5187
netR
dd_30210
delta-3
coupTF-1 dd_33286
zfp-4 dd_29263
colF-11
ephR-1
dd_8655
dd_28888
dd_11341
dd_5147
dd_22514
dd_1296
dd_14596
dd_15903
dachsous
dd_18564
dd_13778
dd_13815
DVB expression verified by FISH
Longitudinal muscle subcluster 1-enriched

S2 Fig

D

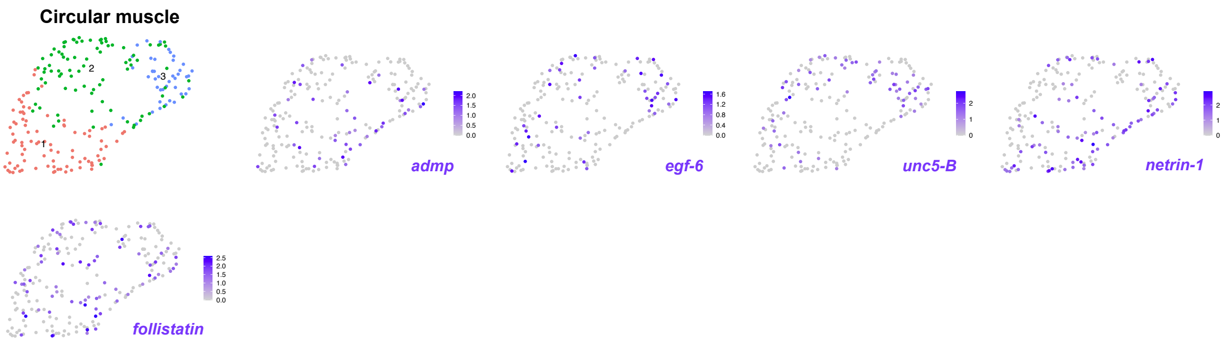

E

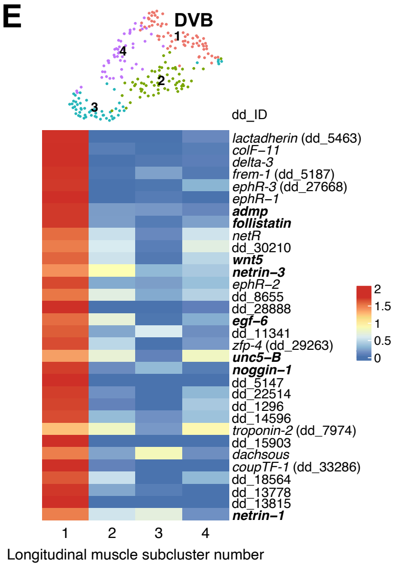

F

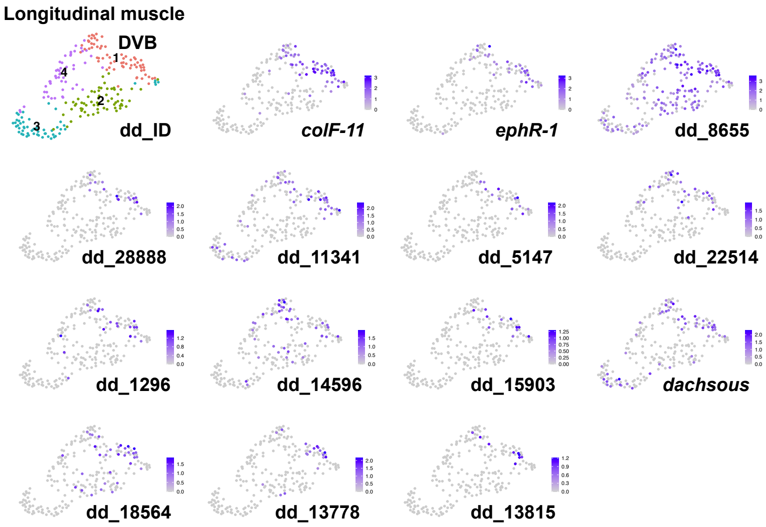

G

**Secreted**	***admp, egf-6, wnt-5, netrin-1, follistatin, noggin-1, netrin-3, lactadherin, frem-1***<br>*colF-11*, dd_8655
**Transmembrane**	***unc5-B, netR, ephR-3, ephR-2, delta-3***<br>*ephR-1*, dd_11341, dd_22514, *dachsous*, dd_13815
**Transcription Factor**	***coupTF-1, zfp-4***<br>dd_28888
**Structural**	***troponin-2***
**Uncharacterized**	**dd_30210**<br>dd_5147, dd_1296, dd_14596, dd_15903, dd_18564, dd_13778

H

dd_ID	PFAM domain description
**dd_8655**	Spond_N (1) / TSP1_spondin (1) / TSP_1 (1) / Kunitz_BPTI (1) / TSP1_CCN (1) / TSP1_CFP_C (1)
**dd_28888**	zf-C2H2 (1) / zf-C2H2_4 (1) / zf-C2H2_6 (1) / zf-met (1)
**dd_11341**	LRR_8 (1) / LRR_5 (1) / LRR_4 (1) / I-set (1) / Ig_3 (1)
**dd_5147**	
**dd_22514**	I-set (1) / Ig_3 (1) / Ig_2 (1) / ig (1) / V-set (1) / V-set_CD47 (1) / DUF6377 (1) / C2-set_2 (1)
**dd_1296**	
**dd_14596**	PDZ (1) / PDZ_6 (1) / PDZ_2 (1)
**dd_15903**	
**dd_18564**	TIMP (1)
**dd_13778**	
**dd_13815**	Trypsin (1) / DUF1986 (1) / Peptidase_S6 (1)

S2 Fig

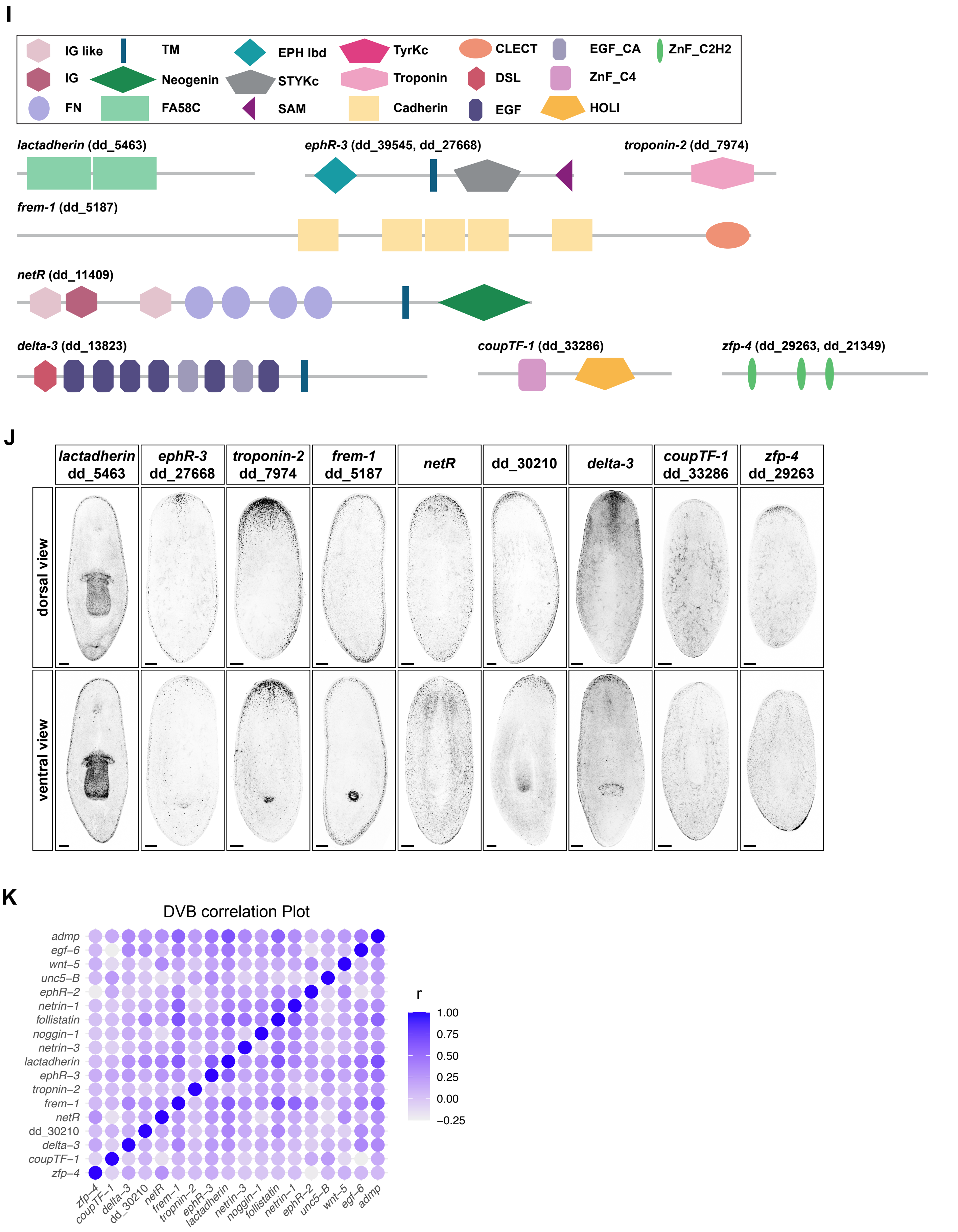
I
IG like
TM
EPH lbd
TyrKc
CLECT
EGF_CA
ZnF_C2H2
IG
Neogenin
STYKc
Troponin
DSL
ZnF_C4
FN
FA58C
SAM
Cadherin
EGF
HOLI
lactadherin (dd_5463)
ephR-3 (dd_39545, dd_27668)
troponin-2 (dd_7974)
frem-1 (dd_5187)
netR (dd_11409)
delta-3 (dd_13823)
coupTF-1 (dd_33286)
zfp-4 (dd_29263, dd_21349)
J
lactadherin dd_5463
ephR-3 dd_27668
troponin-2 dd_7974
frem-1 dd_5187
netR
dd_30210
delta-3
coupTF-1 dd_33286
zfp-4 dd_29263
dorsal view
ventral view
K
DVB correlation Plot
admp
egf−6
wnt−5
unc5−B
ephR−2
netrin−1
follistatin
noggin−1
netrin−3
lactadherin
ephR−3
tropnin−2
frem−1
netR
dd_30210
delta−3
coupTF−1
zfp−4
r
1.00
0.75
0.50
0.25
0.00
−0.25

S2 Fig
