# Supplementary material for: The planarian dorsal–ventral boundary regulates anterior–posterior axis growth and patterning
Source: PLoS Biol. 2025 Nov 11;23(11):e3003482. doi: 10.1371/journal.pbio.3003482 (PMC12629446; doi:10.1371/journal.pbio.3003482)
Supplement: Attachment — Submitted filename: AP-DV Review_reply.docx [file pbio.3003482.s012.docx]

Reviewer #1: In the manuscript entitled 'The dorsal-ventral boundary influences anterior-posterior axis formation in planarians' Maybrun et al. study the role that the DV border could have in allowing the emergence of the AP axis. For doing that they promote the induction of ectopic DV borders, or supress its formation, and they observe a correlation between the existence of a DV border and the expression of anterior markers (anterior PCGs). The authors also analyse at single cell level the DV border and identify different cell clusters. The authors conclude that there is a temporal and hierarchical relationship between the appearance of the DV border and the formation of the AP axis. 
The study demonstrates the importance of the DV border in promoting the growth of a new axis. The essential role of the DV border as a region with the competence to sustain the emergence of a new axis is not new. However, its demonstration in the specific context of planarians and whole-body regeneration—through such an original approach (inducing ectopic DV borders in combination with bcatenin1 inhibition)—is elegant, novel, and highly informative. Additionally, the SC-seq analysis of this specific region provides valuable data for the field.

However, there are major issues regarding some sections of the manuscript and the conclusions that need to be addressed.

We thank the reviewer for their comments, support, and assessment of the work.

Major concerns
1) The more important and general concern is that the requirement of a DV confrontation to promote the growth of a new axis is a well-established mechanism in the field of developmental biology. For instances, the experiments in cricket or axolotl limbs in which grafting a rotated limb promotes the growth of ectopic limbs are well known. The fact that to trigger the regenerative response in planarians it is required the close of the wound and thus the contact between the D and the V domains is also well established. For this reason, 1) this background should be included in the introduction and also in the discussion,

We updated the introduction and discussion to put our work in a broader context. We discussed instances in biology where a DV boundary that promotes growth and patterning of a second axis exists. We suggested that DV boundaries are not present for the early establishment of DV and AP axis pattern in most examined embryos across phylogeny but are present in several contexts in later developmental process where new axes emerge, including during appendage development (e.g. DV boundary of the *Drosophila* wing disc, AER in vertebrate limb development) and during whole-body regeneration in planarians. We hypothesized the following:

“460 One possibility is that outgrowths with patterned axes that emerge later in development, such as appendages, or in adulthood, such as during regeneration, are predominant contexts where boundary-like signaling centers have axis organizing roles. Because these outgrowths need to establish and coordinate axial pattern, they might rely on boundary-like signaling centers with positions determined by embryonic patterning, thereby harnessing existing tissue positional cues to direct new growth.”

Furthermore, we elaborated on contexts in which DV conflicts such as those induced by transplantation are associated with axial outgrowth (including the examples noted by the reviewer):

“94 DV confrontation can promote axis formation in certain patterning contexts in other organisms. In *Drosophila*, for instance, interactions between dorsal and ventral cells in the wing imaginal disc drive wing growth [34, 35]. Genetic manipulations that create an ectopic boundary between dorsal and ventral cells can lead to formation of an ectopic outgrowth of the wing blade.”

“475 For instance, DV misalignment, or conflict, generated by transplantation can lead to supernumerary limbs during development [73] and during regeneration [74, 75]. Transplantations generating positional conflicts of varied types can lead to regenerative outgrowths in diverse organisms, from crickets [76] to salamanders [77] to planarians [3].”

We also note the following:

“99 Whether diverse organisms across the animal kingdom such as those capable of whole-body regeneration have a DVB present in the adult stage that regulates regeneration and the mechanistic roles of such prospective adult DVBs in regenerative contexts are fundamental problems that remain poorly understood.”

“471 Adult cues, like the DVB, might prove to be fundamental to growth and coordination of axial patterning in regenerative outgrowths broadly in animals.”

and 2) all the data presented which refers to the DV axis is in fact related to the DV border (generated by the confrontation of the D and V domain). So, it does not refer to an axis but to a specific domain that acts as a signalling center (in all metazoans).

We agree with the reviewer that our data relates the DVB to a signaling domain at a particular DV position. Where we refer to the DV axis, we aim to use that term in reference to discussion of how patterning of the DV axis establishes the position of the DVB. We argue that there exists a DV axis patterning process (Bmp mediated) that establishes the position of the DVB, as shown in prior work in the field (Reddien, 2007; Molina, 2007). We added reference to this in the introduction:

“91 This DVB location is set by a patterning process on the DV axis involving dorsal Bmp signaling [25, 26].”

We also added an experiment characterizing the location of the *laminB*^+^ DVB relative to molecules that pattern the DV axis:

“145 We used epidermal cells expressing *laminB*, which mark the lateral DV median plane [37, 39], as a reference point for the DVB. At 96 hours post-amputation (hpa), this DVB location is positioned ventral to the dorsally high *bmp4* domain in muscle and is coincident with the known location of *admp* expression in muscle [9, 40, 41] at the DV median plane (Figure 1C, Figure S1H).”

Lastly, we examined the text, endeavoring to make our intent clear regarding our use of the term DV axis:

“41 Our findings reveal a hierarchical model of pattern integration across body axes in which DV patterning is central by producing a DVB with competence to direct formation of large AP axis regions.”

“418 However, we suggest that the primary linkage between patterning of planarian body axis regions during regeneration involves DV patterning processes that form the DVB at the lateral edge of the DV median plane, or body margin. The DVB then regulates AP-axis growth and patterning, restricting growth and anterior positional information expression to the DVB in a Wnt-low environment (Figure 6C-E).”

“1015 Model: DV patterning acts to establish the location of the DVB at the mid-point of the DV axis, at the lateral animal margin. The DVB then serves as a landmark to allow growth and patterning of regions of the AP axis, such as during head regeneration or head formation in a low Wnt environment. This allows the DV axis to control orthogonal AP axis region growth and pattern formation.”

Focusing in planarians, after an amputation, the D and V domains get in touch in the wounded area, and this confrontation of signals triggers the regenerative response, which includes the expression of wound response genes, and PCGs (AP, DV and ML). It is true that the first event is the DV confrontation and thus the emergence of the DV border, but the DV axis was already present in this first stage, when new tissue is still not present. Even more, in the current study the process of regeneration in a wt is not studied, but only the expression of A PCGs in ectopic DB borders (which already have an established DV axis). For this reason, the conclusion illustrated in figure 5E is confusing. In this study it is not demonstrated that there is a hierarchical relationship between both axes, but that A identity (not an AP axis, because to define an axis 2 poles are required) can only be generated in a DV border region.

As noted by the reviewer, the DV axis of a regenerating fragment is a pre-existing pattern, thus existing prior to wound closure. The generic wound response can occur without DV confrontation, for example at incision injuries (Wurtzel, 2015). We added a study of wild-type PCG expression during both anterior and posterior regeneration, as suggested by the reviewer. We included *en face* imaging of PCG expression relative to *laminB*^+^ DVB cells in anterior and posterior blastemas (Figure 1C, Figure S1I). The anterior pole coalesces at the DVB during head regeneration (Oderberg et al., 2017). We noted that both anterior and posterior PCGs are expressed in foci concentrated around the DVB. Interestingly, multiple of these PCGs showed peaked expression on the dorsal and ventral sides juxtaposing the *laminB+* DVB region at anterior- and posterior-facing wounds (Figure 1D, Figure S1J-K). As a control, we showed that the DVB is positioned ventral to the dorsally high *bmp4* domain in muscle and is coincident with the known location of *admp* expression in muscle (Figure 1C, Figure S1H). Thus, because DV axis patterning gives rise to the DVB and anterior and posterior PCGs are expressed near the DVB during regeneration, we propose a hierarchical relationship between DV and patterning of large AP regions during regeneration and homeostatic head formation in a low Wnt environment. The DV axis, through a patterned DVB, informs the location of head and tail outgrowth and patterning.

To address the reviewer’s point regarding our use of the term AP axis when discussing the relationship of the DVB to anterior identity, we modified the text throughout the manuscript to indicate our intended meaning that a large region of the AP axis (the anterior region) can form at the DVB. Examples include the following (as well as those already noted above):

“239 To determine whether the DVB is sufficient to promote formation of an anterior region of the AP axis after *β-catenin-1* RNAi, we endeavored to generate animals with ectopic DVB.”

“287 Together, these results are consistent with a model in which muscle at the planarian DVB is competent to activate anterior PCGs and support formation of an anterior region of the AP axis in a low Wnt environment.”

We also updated the model, now illustrated in Figure 6E, to reflect our conclusion that DV axis patterning determines the location of the DVB and the DVB influences patterning and growth of large regions of the AP axis.

Furthermore, that 'this mechanism enables coordination of orthogonal positional information in the context of regeneration' cannot be concluded in this study, since the orthogonal relationship between bot axis is not studied at all. In summary, the conceptual framework of the study is not the establishment and coordination of the DV and AP axes but rather the role of the DV border as a signaling center or a permissive context in which a second axis can grow. This requires a refocusing of all sections in the manuscript.

We argue that there exists a DV axis patterning process (Bmp mediated) that establishes the position of the DVB (see point above).

Then, as you say, the DVB (positioned by DV patterning) acts as a signaling center or a permissive context in which a large region of a second axis (the anterior of the AP axis) can grow. This results in coordination between DV pattern (setting the location of the DVB) and patterning of large regions of the AP axis (growth at the DVB). We added new data to Figure 1 showing the emergence of anterior and posterior patterning molecules centered near the DVB (see point above). We also looked carefully at our wording throughout the manuscript in light of this comment, to ensure we are being as accurate as possible in the intended meaning of our wording related to this concept.

2) The results and conclusion of the first section are not supported and furthermore they are not relevant for the rest of the findings of the manuscript. Even the meaning of the title is confusing ('Positional information along the AP and DV axes is largely independently maintained'). If the message of this section is that during homeostasis the PCGs are stably maintained, in particular the DV genes in bcatenin1 RNAi animals, this is not observed in Figure 1A. In this figure, bmp4 and ngl-7 expression in the DV border of bcatenin1 RNAi animals is different than in controls. In bcatenin1 RNAi animals bmp4 it's seen in the V view and in controls in the D view. ngl-7 expression in the DV border is not seen in bcatenin1 RNAi animals, but it is in controls.

We thank the reviewer for raising this point. The differences in appearance of some DV PCGs as viewed from the dorsal or ventral side in control and *β-catenin-1* RNAi animals can largely be explained by the ventral curvature of *β-catenin-1* RNAi animals. *admp* expression at the DVB, visible in the dorsal view in control animals, is visible from the ventral view of *β-catenin-1* RNAi because of the downward curvature of the animal. To better show that *admp* is expressed at the DVB in both control and *β-catenin-1* RNAi animals, we cut off the side of the animal and imaged from the side view (Figure S1B). Furthermore, despite differences in *nlg-7* DVB expression between control and *β-catenin-1* RNAi animals viewed from the dorsal or ventral side, imaging of the cut side revealed that *nlg-7* is expressed at the DVB in both conditions (Figure S1B).

Regarding the relevance of the data presented in Figure 1A-B, we endeavored to improve clarity of our wording on the topic:

“116 The AP and DV axes maintain pattern largely independently in homeostasis”

“138 These results point towards the possibility that axis integration might predominantly be accomplished during regeneration or during ectopic head formation in a low Wnt environment, with pattern thereafter largely stably maintained by each axis independently during cell turnover.”

“402 Positional information along planarian body axes is controlled by two largely independent signaling pathways: Wnt for the AP axis and Bmp for the DV axis [10]. This feature is reminiscent of *Drosophila* development, where AP and DV axes are established by largely autonomous, orthogonal patterning systems [61]. Utilization of mechanistically separate molecular pathways to pattern orthogonal tissue axes might represent a common strategy to achieve stable patterns in biology.”

These data demonstrate that large-scale patterns along each axis can be homeostatically maintained during tissue turnover without input from the other axis. This suggests that AP and DV patterns must be integrated during the establishment of axes (i.e. during regeneration and/or ectopic head/tail formation), which is explored in the remainder of the manuscript.

3) While the experiments in which an ectopic DV border is induced do show that A PCGs only appear in these regions, the conclusions after ERK and DV border suppression are not so informative. It is reported in several studies that ERK inhibition blocks the wound response and subsequently any regenerative response. Thus, in the context of ERK inhibition no DV border is generated, because as explained few lines above, the appearance of the DV border signalling center is linked to the regenerative response in general (wound response genes, PCGs of all axis). 
In any case, it is very surprising that even when ERK is not signalling, 9/22 animals show A PCGs expression (see a further comment below). And then in line 239, it is indicated that those regions positive for A PCG do not form overgrowths. This is not indicating that the DV border is required for the growth of an axis, but that ERK blocks any regenerative response, so an overgrowth cannot occur in these conditions.  
The conclusion that 'Removal of the DVB region blocks anteriorization caused by Wnt inhibition or anterior positional information activation in the Wnt-low environment of head regeneration', although it is true, it is misleading, because removal of ERK and the DV border blocks any regenerative signal.

Whereas it is true that ERK signaling promotes wound-induced gene expression that culminates in regeneration (Owlarn et al. 2017), we demonstrated that ERK signaling is not required for the homeostatic formation of overgrowths after *β-catenin-1* RNAi (Figure S4E). This demonstrates that lateral head growth in a low Wnt environment (*β-catenin-1* RNAi) can occur after ERK inhibition. Thus, it is notable that anterior PCG expression associated with overgrowths does not appear when DVB regeneration is blocked by ERK inhibition (Figure 5B). We endeavored to improve our wording regarding this experimental condition in response to this comment:

“307 Erk signaling is required to promote wound-induced signaling that culminates in a regenerative response [60]. Erk signaling was not required, however, for the homeostatic outgrowth that accompanies ectopic head formation after *β-catenin-1* RNAi (Figure S4E).”

“317 However, this expression did not form foci or local outgrowths indicative of head formation (Figure S4F). It is possible that anterior PCG expression can be initiated at low levels in the absence of the DVB, and that the DVB acts to promote elevated and sustained anterior PCG expression that culminates in strong foci.”

“321 Importantly, anterior PCGs were expressed on the uninjured side of both DMSO- and PD- treated animals, where they could form foci, demonstrating that Erk signaling is not generally required for anteriorization during homeostatic turnover (Figure 4B).”

To further support the point that the DVB region is required to promote anterior PCG expression in low-Wnt environments, we now included an additional experiment to support this conclusion. In the prior version of the manuscript, we showed that *bmp4; smad-1* RNAi fragments with one side lateral DVB removed regenerated an anterior PCG expression domain only at the remaining, anterior-facing, intact DVB. We now generated *bmp4; smad-1* RNAi fragments with all lateral DVB removed. In these animals, the DVB did not regenerate and few or no anterior PCG expressing cells were observed at 7dpa (Figure 5C).

4) The relevance of the information obtained after the SC-seq analysis of the DV border is not clear. The different cell populations and the markers identified could be used to analyse more in deep the signalling properties of the DV border. For instances, even when ERK is not signalling, 9/22 animals show A PCGs expression. This result contradicts authors hypothesis, and deserved more attention. Here it is an opportunity to check if in these animals, despite not having laminb, they show expression of other DV markers that could be indicating the presence of a functional DV border.

We now analyzed muscle DVB gene expression in *β-catenin-1* RNAi animals after parasagittal amputation and Erk inhibition (Figure S4G). Despite sporadic anterior PCG expression on the cut side of 9/10 *β-catenin-1* RNAi animals treated with PD, no expression of tested muscle DVB genes (*lactadherin*, *frem-1*) was observed. As noted above, it is possible anterior PCG expression can be initiated at low levels in the absence of the DVB, and the DVB acts to promote sustained and elevated anterior PCG expression that culminates in strong foci.

To address the reviewer’s more general comment, we also utilized genes expressed in muscle cells at the DVB, which were identified in our scRNA-seq analysis, as markers throughout the manuscript (Figure S1N, Figure S3B, Figure S4G, Figure S5A).

We also did further work in analyzing the domain structure and homology of the molecular components that define the DVB region (Figure S2G-I). We now discuss these molecules in the text (line 211-222). The large number of molecules expressed at this central but understudied region of the animal, with their mapped expression domains (Figure 2B-D) and identified muscle subtype expression signatures (Figure S2C), should provide an important resource for the field.

5) Last section 'The DVB has an anterior pole-independent role in the induction of anterior positional information expression after Wnt inhibition' is very confusing. It is published by the same authors of this manuscript that notum expression is downstream of bcatenin1. So, bcatenin1 RNAi animals do not show expression of notum although they regenerate heads. Some days after the RNAi inhibition of bcatenin1 expression of notum can appear, since the dsRNA is not present anymore. This is what could be happening in the animals in Figure 5A. So, the conclusion that the 'DV border plays an anterior pole-independent role in the induction of anterior positional information expression' could be replaced by 'bcatenin1 plays an anterior pole-independent role in the induction of anterior positional information expression', which is another way of saying what was already published by the same authors, that a head can be formed in absence of notum (anterior pole) if bcatenin1 is silenced. It could be that notum is required to inhibit bcatenin1 when an A pole must be specified, but in a very low Wnt environment this is not necessary anymore, and a head is formed, in the absence of the anteror-pole determinant notum. The role of the DV border in this process is not known.

Wnt signaling is required for wound-induced *notum* expression (Petersen and Reddien, 2011). However, *notum* can be detected in the ectopic anterior poles of homeostatic *β-catenin-1* RNAi animals after four feedings of dsRNA (Stückemann et al., 2017). Here, we showed that ectopic *notum* expression at the DVB is not detected until 14-21 days after the initiation of *β-catenin-1* RNAi (Figure 5A). Because ectopic anterior PCGs (*sFRP-1*, *ndl-4*) are detected at the DVB by 7 days post initiation of RNAi (well before the anterior pole was detected), this result suggests that the anterior pole is not required for anterior PCG expression after *β-catenin-1* RNAi. We corroborated this finding using *FoxD* expression as another marker of the anterior pole. Likewise, *FoxD* expression was not detected until 14-21 days after the initiation of *β-catenin-1* RNAi, long after anterior PCGs were induced at the DVB, providing further support for the conclusion that anterior PCG expression became induced without an anterior pole forming. Our *FoxD* RNAi data also showed that anterior PCGs can form at the DVB without an anterior pole following *β-catenin-1* RNAi.

May be silencing of specific markers of the DV border (from the SC-seq data for instances) could be useful to identify specific factors of the DV border which confer properties as a competent region to promote A PCGs expression.

We agree with the reviewer that RNAi of DVB-expressed genes is of interest. We now screened 32 genes identified to be enriched in *myoD*^+^ longitudinal muscle cells at the DVB by RNAi for a functional requirement in anterior PCG expression after *β-catenin-1* RNAi. We also tested the role of *nk4*, a transcription factor that specifies lateral DV muscle (Scimone, 2018). None of the genes tested resulted in an absence of lateral anterior PCG (*sFRP-1*, *ndl-4*) foci formed after *β-catenin-1* RNAi. Data are shown below. Although some genes displayed a weak effect (reduced number of anterior PCG foci as compared to control; *β-catenin-1* RNAi animals), we deemed these effects not substantial enough for clear interpretation. *follistatin* RNAi showed a statistically significant reduction in the number of anterior PCG foci after *β-catenin-1* RNAi. We selected some genes that displayed a weak effect for targeting with combinatorial RNAi. We simultaneously inhibited groups of two or more genes, but no combination tested resulted in an absence of anterior PCG foci after *β-catenin-1* RNAi. Our results are shown below. Future studies, including comprehensive combinatorial RNAi screening, will be required.

Animals were fed dsRNA targeting the indicated gene(s) 4-6 times over 2-3 weeks. Then, animals were fed *β-catenin-1* dsRNA 1-2 times and fixed 10-14 days after the initiation of *β-catenin-1* RNAi. The following dd_IDs correspond to two contigs of a single gene: *ephR-3* (dd_27668) and dd_39545, *ephR-1* (dd_16483) and dd_12794, *zfp-4* (dd_29263) and dd_21349.

Minor concerns
1) In the SC-seq analysis, laminB was used to assign a D or V position. Why laminB is considered the reference of the DV border? Could it be that laminB is in fact in the D or V domain and other genes of the atlas are located in the physical position of the cells in the DV border?

*laminB* has historically been used as an epidermal marker for the DVB and it appears to exist at the point where the dorsal and ventral epidermis meet (Kato et al., 1999;Tazaki, 2002). In the scRNA-seq data analysis, we focused our study on muscle and used known DVB genes expressed in muscle to identify a DVB muscle cell population. We analyzed by FISH the expression of genes identified through this analysis and used *laminB* simply as a reference point for each gene, allowing us to ascertain the relative positions of genes to one another by use of a common landmark. In principle, an alternative DVB gene could have been used as a landmark, but *laminB* proved an effective landmark that can be robustly detected with FISH. We also added an experiment to show the position of *laminB*^+^ cells relative to the expression of DV patterning molecules *bmp4* and *admp* to further support our choice of this reference marker (Figure 1C, Figure S1H). We edited the text to reflect that *laminB* simply serves as a reference point.

“145 We used epidermal cells expressing *laminB*, which mark the lateral DV median plane [37, 39], as a reference point for the DVB. At 96 hours post-amputation (hpa), this DVB location is positioned ventral to the dorsally high *bmp4* domain in muscle and is coincident with the known location of *admp* expression in muscle [9, 40, 41] at the DV median plane (Figure 1C, Figure S1H).”

2)The existence of 2 axis is linked to the appearance of the midline. If the study aims to analyse the relationship between the DV and the AP axis (which is not the current form), considering the midline is essential. Not only during regeneration of a wt animal (Oderberg et al. 2017), also in bcatenin1 RNAi animals each ectopic head always show expression of slit in the midline (Stückemann et al. 2017). Rojo-Laguna et al. 2019 (a missing reference) also show that all overgrowths have DV border (if-b) but they only have AP identity when they recover the midline (slit). 
Another context that demonstrates the requirement of the midline in addition to the DV border is that when a wound is generated near the head of a wt animal, which has already a low Wnt environment, it does not promote the expression of A PCGs and the formation of an ectopic head. In wt animals it only occurs in the DV border that crosses the midline region. 
The analysis or at least a in deep discussion on the role of the midline is required.

We added discussion on the midline in relation to AP patterning:

“423 The linkage of AP-axis patterning to the position of the DVB is also intimately connected to the ML axis. The anterior pole coalesces specifically at the intersection of three landmarks (the DVB, the midline, and at anterior-facing wounds) during head regeneration [33]. Transplantation of tissue with reversed DV polarity can lead to the formation of an ectopic head and/or tail in resultant outgrowths [37] [62]. Rojo-Laguna, 2019 show that in AP polarized outgrowths the midline marker *slit* becomes patterned along the emergent ML axis, whereas outgrowths that lack AP polarity display aberrant expression of *slit* [62]. After *β-catenin-1* RNAi, ectopic midlines are established in ectopic heads that emerge from the animal lateral edge [19].”

3) When performing grafts that induce new DV confrontations there appear overgrowths, which can have different shapes and identities. For instances, Rojo-Laguna et al. 2019 show that the inclusion/exclusion of the VNCs gives rise to different overgrowths shape and identities.  It is not clear in the present manuscript how the grafts were performed (a more detailed procedure is required) neither the identity of the outgrowths in the different contexts.
In lines 184-185- the authors describe that the outgrowths in Figure 3 have the AP character typical of the location of the transplant. Does it mean that if they are posterior to the pharynx, they are P, and if they are in the prepharyngeal region they are A? Then, the overgrowth in Figure 3C has P identity? This should be demonstrated with the corresponding markers if it is stated in the text. In addition, the outgrowths produced after the DV confrontation in control and bcatenin1 RNAi animals appear in D and in V regions. Only the D ones have A identity in bcatenin1 RNAi animals? What about the identity of the V outgrowths? The A and P identities of the outgrowths should be clarified with markers when required as well as a better explanation of the procedures and results.

We updated the methods section to include additional details on the transplant procedure:

“595 A ~250µm piece of tissue at the midline posterior to the pharynx was excised from a recipient animal, and the animal was allowed to recover in Holtfreter’s solution for 2 minutes. For D-V transplants, after a second incubation in chloretone, donor tissue (from a matched AP and ML location) was transplanted into the recipient with reversed DV polarity.”

We now included a molecular characterization of outgrowth identity before *β-catenin-1* RNAi. Outgrowths in the post-pharyngeal region expressed *wntP-2*, indicating that they have a posterior identity (Figure S3C). We focus on dorsal outgrowths in the manuscript because they were more readily attained. We studied ventral outgrowths as suggested and found that they adopted anterior identity (*sFRP-1*, *ndl-4*^+^) after *β-catenin-1* RNAi (Figure S3D).

4) The analysis of the expression of DV and AP (and ML) PCGs in controls compared to different RNAi conditions would be very informative in regenerating animals if the purpose is to understand the hierarchy of the body axis during regeneration. For instances, do DV or DV border genes appear earlier than AP genes? How does the RNAi of DV genes affects the expression of AP genes? And the other way around?

We studied the order of onset of muscle DVB gene expression versus anterior PCG expression during head regeneration. The onset of anterior PCG expression occurs between 24-48 hours post-amputation (hpa) (Gurley, 2010; Scimone, 2017). At 36 hpa, cells expressing muscle DVB genes, including those identified in this study, were detected at the edge of head blastemas, when anterior PCGs showed robust, focused expression (Figure S5A). This is consistent with the DVB playing a role in the promotion of elevated anterior PCG expression and foci formation.

We tested by RNAi whether any of the *myoD*^+^ subcluster 1-enriched genes were individually required for anterior PCG expression or ectopic head formation after *β-catenin-1* RNAi and none robustly were, as noted above. This does not exclude a role for any of these DVB genes in blastema PCG activation. Future additional RNAi screening will be important to resolve this, in light of potential redundancy, but we deemed this beyond the scope of this review period given no immediate hits were obtained.

Reviewer #2: The manuscript provides novel evidence that the dorsoventral boundary (DVB) in planarians is necessary and sufficient for the expression of anterior genes and subsequent head formation, especially under conditions that elicit the formation of ectopic heads via reduced canonical Wnt signaling.  The authors use both surgical and molecular manipulations to create or eliminate DVBs and show that these can act as foci for anterior gene expression.  They further provide evidence that anterior gene expression does not rely upon the formation of anterior pole cells.  While the study does not investigate the molecular mechanisms linking the DVB to anterior gene expression, the data is largely sound and well-presented.  

The authors also uncover new genes expressed along the DVB which will be valuable for future studies.  But the new DVB genes were not utilized as markers in this study, and their roles in DVB formation or maintenance were not tested.

We thank the reviewer for their comments, support, and assessment of the work.

I do have a few concerns on specific results, or on the presentation of results, that I would like to see addressed.

1) Anterior markers after beta-catenin-RNAi.  The authors suggest throughout the manuscript that there is a tight correlation between the DVB and the ectopic expression of anterior makers after knockdown of canonical Wnt signaling by beta-catenin-RNAi.  However, in Figure S1A the ectopic sFRP-1 induced by the RNAi was not limited to the DVB.  Although it was stronger in foci near the DVB, there was also scattered ectopic expression throughout the animal that appears every bit as strong as that near the head.  I think this needs to be briefly discussed, and the authors need to be clear on what level and concentration of expression they are referring to elsewhere in the manuscript.

The control *sFRP-1* expression domain includes the head tip, as well as a midline stripe that extends slightly posterior to the head tip. Inhibition of Wnt signaling through *β-catenin-1* RNAi lead to the formation of ectopic foci of anterior PCG expression at the DVB in the posterior of the animal (Figure S1A). We agree with the reviewer, however, that *sFRP-1* is also detected ectopically in scattered cells in the body.

We addressed this point in the text:

“176 Although anterior PCG (*sFRP-1*, *ndl-4*) expression was concentrated at the DVB after *β-catenin-1* RNAi, sporadic cells expressing anterior PCGs were also detected dispersed in the animal (Figure S1A). Hereafter, we analyze specifically the anterior PCG expression that occurred in a concentrated manner at the DVB in *β-catenin-1* RNAi animals.”

We also included images showing the concentration of ectopic expression of an additional anterior marker, *ndl-4*, at the DVB after *β-catenin-1* RNAi. (Figure S1A).

2) "111 In agreement with findings in Clark, 2023 [26], we found that the domain of
112 wnt1+ cells (the posterior pole) was expanded after 45 days of bmp4 RNAi (Figure 1B,
113 Figure S1D)."
To my eye the expression looks ectopic rather than expanded, as it is largely limited to a mid-posterior region that does not extend to the domain of normal expression at the posterior pole. However, this statement, and the bars in Fig. 1B, imply continuous expression from the posterior to mid-posterior.

We added an enlarged inset showing posterior expression of *wnt1* after *bmp4* RNAi (Figure S1E)*.*

3) "236 In some cases (n=9/22), reduced anterior PCG
237 (sFRP-1 and ndl-4) expression was detected on the injured side of PD-treated β-
238 catenin-1 RNAi animals, despite no regeneration of laminB cells at the DVB."
The statement is confusing: "reduced" compared with what?  I think the authors mean "low-level expression was detected".

The level of anterior PCG expression detected on the injured side of PD-treated *β-catenin-1* RNAi animals is lower compared to the level detected in DMSO-treated animals. We have now updated the text, avoiding the use of “reduced”, to remove this point of confusion.

“314 In some cases (n=9/22), anterior PCG (*sFRP-1, ndl-4*) expression was detected on the injured side of PD-treated *β-catenin-1* RNAi animals, despite no regeneration of epidermal DVB (*laminB*) (Figure S4F) or muscle DVB (*lactadherin*, *frem-1*) gene expression (Figure S4G).”

4) Fluorescence images and PD treatment- The authors show multicolor fluorescence images that are, on my screen, rather dark, and the overlapping domains of expression are never presented as single-channel images.  This makes it very difficult to judge the purple channel anterior marker data, either when it is low or when it overlaps strong green staining.  I think it is important to show the single channel data on its own.

The issue is especially critical for the PD treatment data of Figures 4B and S5D-E.  First, when viewed without the green channel Fig S5D shows that MEK inhibition via PD treatment alone greatly increases anterior gene expression in all the remaining DVB cells.  If this is not a mistake with the authors' image processing this is an important (and confounding) result that the authors need to address. The authors are using DB treatment as way of blocking regeneration of the DVB after surgical removal, and in 4B and S5E say this largely blocks expression of anterior genes after beta-catenin-RNAi in the region where the DVB was removed.  However, if DB treatment on its own causes anterior-gene expression along the DVB, then the RNAi experiment seems superfluous.

The magenta signal the reviewer refers to is background (i.e., outside the epidermal layer and non-overlapping with DAPI) (see figure below for a zoomed-in view of a small z-stack at the lateral edge after PD treatment that illustrates this point). By contrast, the true anterior PCG (*sFRP-1*, *ndl-4*) signal detected is in planarian muscle, internal to the epidermis (*laminB*^+^).

We now included single-channel images which show that PD treatment does not induce the expression of anterior PCGs at the DVB (Figure S4D). To avoid any confusion, we exchanged the image of a PD-treated animal in Figure S4D. The updated image reflects a representative animal with less background signal.

Additionally, the authors claim to see two different classes of results in S5E, but the differences in intensity of the anterior genes on the left DVB of each figure are not that obvious when viewed without the green channel.

We included single-channel images and zoom-ins which show very few *sFRP-1, ndl-4*^+^ cells are detected in 12/22 *β-catenin-1* RNAi animals treated with PD after parasagittal amputation (Figure S4F). By contrast, in 9/22 animals, anterior PCG expression is detected on the cut (left) side of the animal.

Minor note: placing the 12/22 over the yellow detail box in 4D, and then having the magnified version of the box say 22/22, is confusing.

We adjusted the position of the experimental quantification to communicate our result that 12/22 animals displayed little anterior PCG expression on the injured (left) sides of the *β-catenin-1* RNAi animals which had DVB removed. All 22 animals displayed ectopic anterior PCG expression at the intact DVB (right side of the animal) (Figure 5B). We modified the figure legend to include this quantification:

“993 Treatment with the MEK inhibitor PD blocks lateral anterior PCG (*sFRP-1*, *ndl-4*) expression after *β-catenin-1* RNAi and DVB removal (n=12/22). Anterior PCGs are expressed in foci at the intact DVB on the uninjured side of PD-treated *β-catenin-1* RNAi animals (n=22/22).”

5) The section about the bmp4;smad-1 RNAi experiments could be clarified. The 3/9 vs 6/9 of Fig. 4C is never referred to in the text or legend.

We clarified our wording at several points to make this experiment description clearer. Here, we repeated the experiment and included updated quantification in the text:

“345 In some cases, anterior PCGs were expressed on only one side of the fragment at the DVB (left side (n=8/26) or right side (n=10/26)). In other cases, two foci of anterior PCG expression were observed, with one near the left DVB and one near the right DVB (n=8/26).”

The "By contrast" sentence about the lack of regeneration of the DVB from the RNAi-treated fragments seems out of place, as the next two sentences refer to fragments that had their DVBs intact.

*bmp4; smad-1* RNAi fragments did not regenerate the DVB at wounds. This contrasts with control animals, which regenerated *laminB*^+^ cells at both anterior- and posterior-facing wounds. In the timescale of our experiment, the intact, lateral DVB (left and right side of the animal) of post-pharyngeal fragments is retained after *bmp4; smad-1* RNAi. We edited the text to make this point clearer by separating our discussion of post-pharyngeal fragments with intact lateral DVB and post-pharyngeal fragments with one lateral DVB side removed:

“337 We generated control or *bmp4; smad-1* RNAi post-pharyngeal fragments. Control post-pharyngeal fragments expressed anterior PCGs (*sFRP-1*, *ndl-4*) at the midline of the anterior-facing wound (AFW) near the regenerated DVB (*laminB^+^*) (Figure 5C).”

“340 By contrast, *bmp4; smad-1* RNAi post-pharyngeal fragments did not regenerate the DVB at their wounds, leaving the fragments with only lateral domains of DVB (pre-existing prior to injury). Anterior PCGs were expressed at the AFW of these *bmp4; smad-1* RNAi fragments but this expression only occurred at the lateral fragment edge near the pre-existing, intact DVB in all cases (n=26/26) (Figure 5C).”

“350 We next generated *bmp4; smad-1* RNAi post-pharyngeal fragments and also removed one side of lateral DVB.”

And for: 
"In bmp4;smad-1 RNAi fragments with
262 one DVB side removed, anterior PCGs were expressed at the AFW in most cases
263 (n=9/10). However, anterior PCG expression was restricted to the lateral edge only near
264 the remaining DVB in all cases (n=9/9) (Figure 4C)."
the authors do not say what happens in the 1/10 fragment; no PCG expression?

We repeated the experiment and included updated quantification. 3/34 animals did not express anterior PCGs at a level detectable by FISH. We added wording to indicate this outcome:

“351 Anterior PCGs were expressed at the AFW in most cases (n=31/34; n=3/34, anterior PCGs were not detected).”

And is the last sentence referring to the 9 of that 10 that had the DVB removed, or to the 9 with the DVB intact?

We updated the sentence, now in line 352, to read:

“352 Notably, in these 31 animals, anterior PCG expression was restricted to the lateral edge only, exclusively near the side with remaining DVB (n=25/25, right DVB removal and n=6/6, left DVB removal) (Figure 4C).”

It refers to the 31/34 animals that expressed anterior PCGs near the AFW after one side of lateral DVB was removed from a post-pharyngeal fragment.

Note that we also added an additional experiment to this section. We now generated *bmp4; smad-1* RNAi fragments with all lateral DVB removed. In these animals, the DVB did not regenerate and few or no anterior PCG expressing cells were observed at 7dpa (Figure 5C). This supports the conclusion that the DVB is required for the emergence of an anterior PCG expression domain in regeneration.

Reviewer #3: This is a very nice report that advances our understanding of the relationship between D/V patterning and A/P specification in Planaria.  It builds on previously published work from the Agata lab from a quarter of a century ago, as well as more recent work indicating a central role for BMP/SMAD signaling in controlling the D/V axis.  While it was known previously that ectopic head formation caused by RNAi of beta-catenin occurs at the lateral periphery of these Planaria, the authors use scRNAseq to define these cells at the D/V boundary (DVB).  They go on to show through a combination of grafting (ala Agata and colleagues) and compound RNAi experiments (making very nice use of the temporal control of administering the dsRNA - a feature that might be unique to Planaria) that the DVB cells are sufficient and necessary for ectopic head formation.  They also do a careful temporal analysis to support a model where DVB expression at the site of ectopic heads precedes
that of the anterior pole.  

I can't identify any significant weakness in this story.  Also, I believe this is a significant enough advance in our understanding of how two axes coordinate during regeneration to merit publication in PLOS Biology.

We thank the reviewer for their comments, support, and assessment of the work.
Editor Summary

As you will see, the reviewers are generally positive about your study and think it is well done and presented. Reviewer #1 raises concerns with the conceptual framing of the study and notes that the manuscript should be refocused on the role of the DV border as a signaling center. The reviewer also notes several cases where the conclusions are not adequately supported by the data. In addition, Reviewer’s #1 and #2 note that the DVB markers identified in the scRNA-seq dataset were not used to provide a more in depth analysis of their signaling roles at the DV border. After discussions with the Academic Editor, we encourage you to strengthen the study along these lines and use the gene expression dataset to provide some preliminary insights into their roles in DVB formation or maintenance.
