# Supplementary material for: The planarian dorsal–ventral boundary regulates anterior–posterior axis growth and patterning
Source: PLoS Biol. 2025 Nov 11;23(11):e3003482. doi: 10.1371/journal.pbio.3003482 (PMC12629446; doi:10.1371/journal.pbio.3003482)
Supplement: Attachment — Submitted filename: AP-DV_Reviews_Final.docx [file pbio.3003482.s013.docx]

Reviewer #1: In the revised version of the manuscript, now entitled "The planarian dorsal-ventral boundary regulates anterior-posterior axis growth and patterning", Maybrun et al. have addressed most of the main concerns. However, the SC-seq data of the DVB remain disconnected from the rest of the experiments. These data could potentially serve as the basis for functional studies in future work. 
Before publication, the authors should carefully review the numbering of the figures cited in the text, as some of them are incorrect.

We thank the reviewer for their assessment of the work. We have corrected all figure callouts in the text.

Reviewer #2: Many thanks the authors, the manuscript has been improved, and my difficulties have been largely dealt with.  I think this should be published, although I have a couple minor points I'd like the authors to consider.

We thank the reviewer for their assessment of the work.

1) There are now two places in the manuscript where the authors distinguish between scattered "sporadic" expression of anterior makers and true anterior foci.  The first is in their added language about the results of bcat-RNAi.  The second is in their 9/22 outcomes with less focused anterior maker expression after cutting, bcat-RNAi and PD treatment to block DVB regeneration.  In the latter, the authors give a fuller possible explanation, low level anterior induction that does not rise to the level or organizing foci.  It might be helpful to have some of that language in the first discussion of the simple bcat-RNAi result, and try and define their criteria a bit.

We modified the text and Figure S1A legend describing the homeostatic *β-catenin-1* RNAi phenotype to better define our criteria for anterior PCG expression in foci at the DVB.

“177 Although anterior PCG (*sFRP-1*, *ndl-4*) expression was concentrated in foci at the DVB after *β-catenin-1* RNAi, sporadic cells expressing anterior PCGs were also detected dispersed (not in foci) in the animal (Figure S1A).”

“1032 Foci containing multiple anterior PCG^+^ cells were apparent at the DVB (foci typically had >10 cells). Scattered anterior PCG^+^ cells were also present in the animal.”

2) A couple remaining clarifications about numbers:

Does the 18/21 in Figure S4D mean that in 3/21 cases the DVB still formed after cutting and PD treatment?

In 18/21 animals, regeneration of the DVB was blocked by PD treatment. In 3/21 animals, a patch of *laminB*^+^ DVB was present after parasagittal amputation and PD treatment. This indicates that DVB was either incompletely removed by the cut or partially regenerated despite PD treatment. We added text to the figure legend to indicate this outcome.

“1091 In 3/21 animals, a patch of *laminB*^+^ DVB was present after parasagittal amputation and PD treatment, indicating incomplete DVB removal or partial DVB regeneration.”

In the Fig. 5B/S4F data, if 12/22 don't have and 9/22 do have anterior gene expression after cutting, bcat-RNAi and PD treatment, what did the remaining 1/21 do?

One of the 22 animals had a patch of *laminB*^+^ DVB remaining on the cut side after PD treatment. We added text to the figure legend to indicate this outcome.

“1099 One animal had a patch of *laminB*^+^ DVB present at the wound (n=1/22), indicating incomplete DVB removal or partial DVB regeneration.”
